# Rates of perinatal environment risk factors in schizophrenia patients with higher and lower schizophrenia polygenic risk scores

**DOI:** 10.1192/j.eurpsy.2024.611

**Published:** 2024-08-27

**Authors:** M. Alfimova, M. Gabaeva, T. Lezheiko, V. Plakunova, V. Golimbet

**Affiliations:** ^1^Clinical Genetics Laboratory, Mental Health Research Center, Moscow, Russian Federation

## Abstract

**Introduction:**

Understanding the relations between genetic (G) and environmental (E) factors in the development of schizophrenia is important for psychosis prevention. These relations may vary from G x E correlations to G x E interactions and independent additive effects of genetic load and environment. The G x E interactions mean that genetic variants associated with schizophrenia make an individual vulnerable to specific environmental exposures thus enhancing the risk of disease manifestation in those who possess such genetic variants. In the case of independent effects, environmental exposure might serve as the main cause or an additional to genetic load external trigger which is needed for the illness development. Thus, the rate of independent environmental risk factors is expected to be higher in patients with a lower genetic liability to schizophrenia.

**Objectives:**

The study aimed to confirm this hypothesis by comparing schizophrenia patients with higher and lower polygenic risk scores for schizophrenia (SZ-PRS) on the rate of urbanicity, winter birth and obstetric complications (OC), as previous data suggested their independence from the genetic burden of the disease.

**Methods:**

SZ-PRS were calculated for 861 patients with schizophrenia spectrum diagnoses (ICD-10, F2), predominantly of Slavic decent, based on the latest GWAS. For patients comprising the highest and lowest SZ-PRS deciles, information on the environmental risk factors was extracted from medical records. Each environmental factor was coded as present/absent. The presence were defined as being born in the most urban environment (a city’s population > 5 million), in winter months and having at least one OC from a predefined list (Alfimova *et al.* Int J Mol Sci 2022; 23: 12629). In addition, hypoxia/asphyxia, and low birth weight were analyzed separately. Polyenvirommental risk scores (PERS) aggregating the three factors were calculated using natural logarithms of the odds ratios (OR) from an umbrella review (Radua *et al.* World Psychiatry 2018; 17: 49-66). Logistic regression adjusted for ancestry-related principal components, demographic, and technical variables was applied to compare the SZ-PRS deciles on each factor and PERS.

**Results:**

None of the factors alone or PERS predicted SZ-PRS decile membership.

**Conclusions:**

The results did not support the hypothesis. Future research needs reliable data on the frequency of the studied factors in the general population where the patients come from.The study was supported by the Russian Science Foundation, grant no. 21-15-00124.

**Disclosure of Interest:**

None Declared

